# Supplementary material for: Multi-view Co-training for microRNA Prediction
Source: Sci Rep. 2019 Jul 29;9:10931. doi: 10.1038/s41598-019-47399-8 (PMC6662744; doi:10.1038/s41598-019-47399-8)
Supplement: Supplementary file 1 — Supplemental Materials [file 41598_2019_47399_MOESM1_ESM.docx]

# Multi-view Co-training for microRNA Prediction

## Supplemental Information

Mohsen Sheikh Hassani^1^, James R. Green^1^*

^1^Department of Systems and Computer Engineering,
Carleton University, Ottawa, Ontario, Canada.

Supplementary Figure 1. Leraning curve for 15 iterations of multi-view co-training for human. No significant increase in performance is observed beyond 11 iterations, justifying this parameter selection in all experiments.

*hsa*: Expression Sequence

*mmu*: Expression Sequence

*dme:* Expression Sequence

Supplementary Figure 2. Final precision-recall curves for classification using co-training, single-view (self-training), passive learning, and no learning for expression and sequence-based views on the human, mouse and fruit-fly data sets. In all plots, the y-axis represents precision while the x-axis is recall. Optimal performance is in the top-right corner of the plot.

*bta:* Expression Sequence

*gga*: Expression Sequence

*eca*: Expression Sequence

Supplementary Figure 3. Final precision-recall curves for classification using co-training, single-view (self-training), passive learning, and no learning for expression and sequence-based views on the cow, chicken and horse data sets. In all plots, the y-axis represents precision while the x-axis is recall. Optimal performance is in the top-right corner of the plot.

**Supplemental Table 1**. Means and standard deviations (in parentheses) for final iterations of each view, for each approach, for all six species. These results correspond to Table 2 in the main manuscript.

| **Data set** | **Expression-based classifier’s average AUPRC** | | | | **Sequence-based classifier’s average AUPRC** | | | |
| --- | --- | --- | --- | --- | --- | --- | --- | --- |
|  | No learning | Passive learning | Self-training | Co-training | No learning | Passive learning | Self-training | Co-training |
| *hsa* | 0.597  (±0.03) | 0.672 (±0.002) | 0.770 (±0.002) | 0.779 (±0.001) | 0.344  (±0.03) | 0.616 (±0.003) | 0.720  (±0.002) | 0.761  (±0.001) |
| *mmu* | 0.714  (±0.01) | 0.708 (±0.002) | 0.883 (±0.001) | 0.955 (±0.001) | 0.822  (±0.01) | 0.887 (±0.002) | 0.895  (±0.001) | 0.912  (±0.001) |
| *dme* | 0.810  (±0.02) | 0.883 (±0.001) | 0.886 (±0.001) | 0.901 (±0.001) | 0.864  (±0.02) | 0.909 (±0.002) | 0.912  (±0.001) | 0.921  (±0.001) |
| *bta* | 0.778  (±0.02) | 0.827 (±0.002) | 0.815 (±0.002) | 0.865 (±0.001) | 0.357  (±0.03) | 0.654 (±0.002) | 0.732  (±0.002) | 0.809  (±0.001) |
| *gga* | 0.925  (±0.01) | 0.923 (±0.002) | 0.946 (±0.001) | 0.964 (±0.001) | 0.893  (±0.02) | 0.894 (±0.002) | 0.911  (±0.002) | 0.927  (±0.001) |
| *eca* | 0.921  (±0.01) | 0.941 (±0.001) | 0.942 (±0.001) | 0.958 (±0.001) | 0.875  (±0.02) | 0.884 (±0.002) | 0.932  (±0.001) | 0.961  (±0.001) |
